# Methods to Determine Chain-Breaking Antioxidant Activity of Nanomaterials beyond DPPH^•^. A Review

**DOI:** 10.3390/antiox10101551

**Published:** 2021-09-29

**Authors:** Andrea Baschieri, Riccardo Amorati

**Affiliations:** 1 Istituto per la Sintesi Organica e la Fotoreattività, Consiglio Nazionale delle Ricerche (ISOF-CNR), Via P. Gobetti 101, 40129 Bologna, Italy; andrea.baschieri@isof.cnr.it; 2 Department of Chemistry “G. Ciamician”, University of Bologna, Via S. Giacomo 11, 40126 Bologna, Italy

**Keywords:** nanomaterial, antioxidant, radicals, autoxidation, nanoantioxidants, oxygen, assays, reactive oxygen species, ROS, catalysis

## Abstract

This review highlights the progress made in recent years in understanding the mechanism of action of nanomaterials with antioxidant activity and in the chemical methods used to evaluate their activity. Nanomaterials represent one of the most recent frontiers in the research for improved antioxidants, but further development is hampered by a poor characterization of the ‘‘antioxidant activity’’ property and by using oversimplified chemical methods. Inhibited autoxidation experiments provide valuable information about the interaction with the most important radicals involved in the lipid oxidation, namely alkylperoxyl and hydroperoxyl radicals, and demonstrate unambiguously the ability to stop the oxidation of organic materials. It is proposed that autoxidation methods should always complement (and possibly replace) the use of assays based on the quenching of stable radicals (such as DPPH^•^ and ABTS^•+^). The mechanisms leading to the inhibition of the autoxidation (sacrificial and catalytic radical trapping antioxidant activity) are described in the context of nanoantioxidants. Guidelines for the selection of the appropriate testing conditions and of meaningful kinetic analysis are also given.

## 1. Introduction

Autoxidation, or peroxidation, is the spontaneous reaction of organic molecules with O_2_ that leads to the formation of peroxides, epoxides, aldehydes and ketones as well as to simpler breakdown fragments or to insoluble polymers. These products have usually unwanted characteristics, such as bad smell (i.e., the rancid odour of oxidized lipids), chemico-physical characteristics that are very different from those of the non-oxidized substrate and, most importantly, toxic effects due to their strong electrophilicity. Although autoxidation occurs in disparate organic substrates including plastic, lubricants, fuels, edible oils, meat, cosmetics and cellular membranes, in all cases the reaction occurs via a radical chain mechanism involving carbon and oxygen centered radicals [1]. The importance of stopping or delaying the autoxidation is of paramount importance for many industrial applications and is still attracting the interest of many researchers. Antioxidants can be defined as molecules able to slow down the oxidative radical chain, therefore preventing the damage that can be caused to oxidizable substrates by the effects of oxygen. Antioxidants comprise an incredibly vast and heterogeneous family of small organic and inorganic molecules, as well as macromolecules and enzymes, all capable of interfering at different stages of the autoxidation reaction.

Antioxidants can be divided into two groups, depending on their mechanism of action: (i) *preventive antioxidants*, that interfere with the initiation process by retarding or stopping the initial formation of radical species and (ii) *chain-breaking antioxidants*, that slow down the autoxidation by competing with the propagation reactions; that is, they react with radicals faster than the oxidizable substrate. In addition to these direct antioxidants, compounds that do not themselves possess antioxidant activity, but can stimulate and increase the efficacy of the endogenous antioxidant defences in biological systems, are usually classified as *indirect antioxidants* [2].

Although antioxidants have been known from a long time, the research of improved natural or synthetic antioxidants is still a hot topic because of their important practical implications. Antioxidants may suffer from low stability under O_2_ and in biological systems they can be degraded before reaching the target sites, or they can have adverse health effects that limit their use [3,4]. Sometimes, it would be desirable removing antioxidants from the homogeneous system to which they were added, after their effect [5].

In this context, nanotechnology has opened up new possibilities to exploit the nearly infinite and innovative properties of nanomaterials, possibly in combination with some conventional natural or synthetic compounds, with the aim of obtaining pioneering “nanoantioxidants” with enhanced properties [6,7,8,9]. More interestingly, many categories of nanoantioxidants have potent radical scavenging and quenching capacities in combination with interesting mechanical, optical, magnetic, catalytic, and optical properties increasing their potential practical use [10,11].

**Figure 1:**
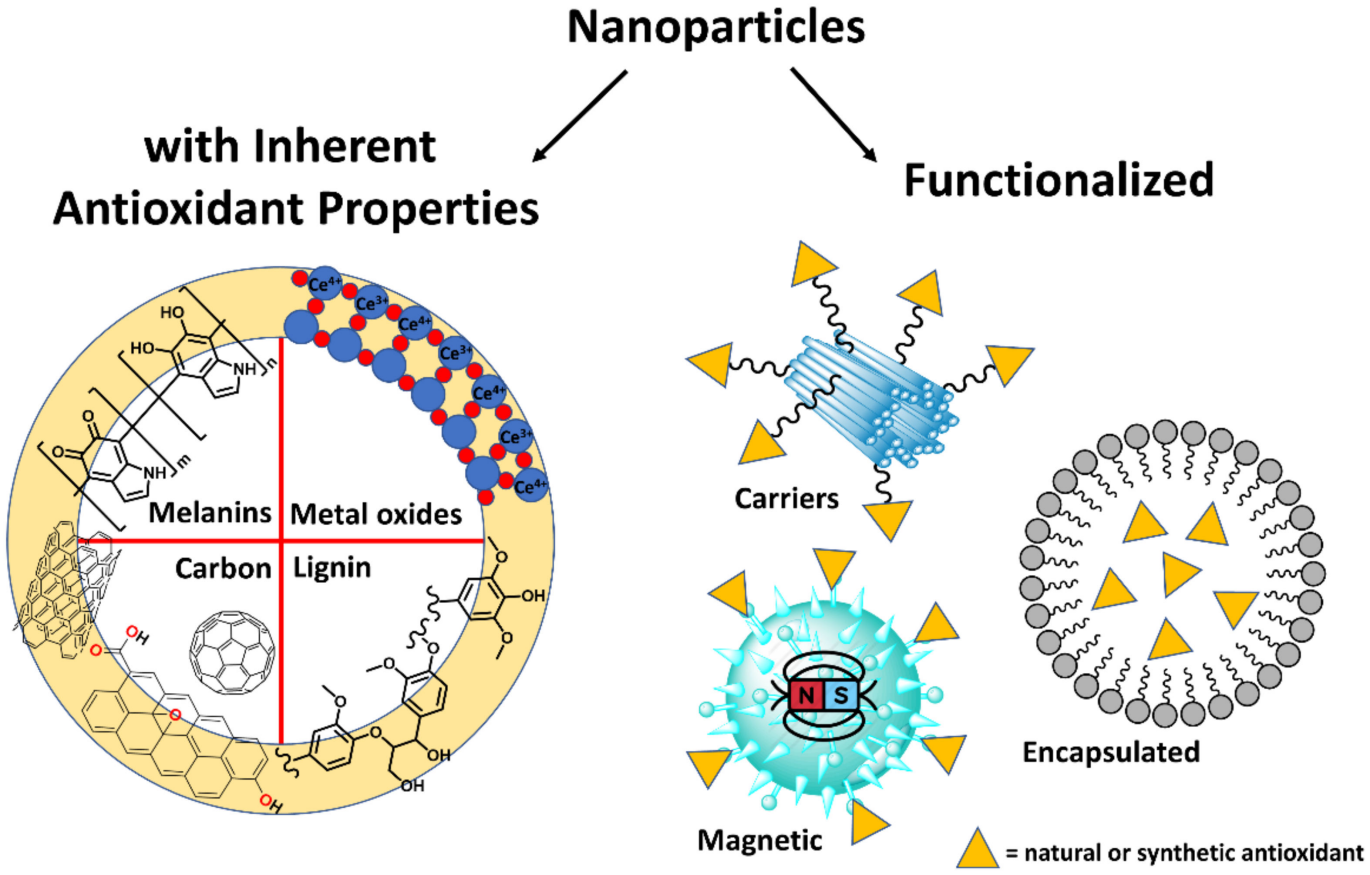
Nanoparticles with antioxidant activity: “nanoantioxidants”.

Antioxidant nanoparticle can be classified into two broad categories: (i) nanoparticles with inherent antioxidant properties; (ii) inert nanoparticle functionalized with antioxidants. The latter can be of very different types, such as a core with antioxidants covalently bound on the surface (e.g., in the case of magnetic nanoantioxidants), or passive carriers able to deliver and release small-molecular antioxidants (e.g., nano-encapsulated, nanotubes or mesoporous materials) [12,13,14,15,16] (Figure 1).

We believe that this classification is still useful to inspire the research in this field, as it is demonstrated by the selected recent examples reported in the next section. However, while there is an enormous effort in the synthesis and physical characterization of these new materials, much less is known regarding the mechanism of radical trapping and the ability to stop the autoxidation of easily oxidizable substrates. In the Section 5 we will show how these two fundamental properties can be easily measured by studying the inhibition of the autoxidation of standard substrates.

## 2. Recent Examples of Nanomaterials Used for Their Antioxidant Activity

### 2.1. Inherent Antioxidant Nanoparticles

The most common nanoparticles (NPs) with intrinsic antioxidant properties are based on metal oxides. CeO_2_ nanoparticles (CeNPs) have been used as efficient antioxidants thanks to the ability of the metal to cycle between Ce^3+^ and Ce^4+^ ionic states. The lack of oxygen atoms on the surface of the nanoparticle leads to the presence of electrons on the 4f orbitals of some cerium atoms. Not all cerium (IV) atoms are converted to cerium (III), thus, Ce^3+^ and Ce^4+^ exist simultaneously on the surface to form a redox pair [17].

Cerium oxide nanoparticles have been widely used for solid oxide fuel cells, electrochromic thin films, sensors, catalysts, and biomedical materials [18,19]. In 2019, Shi and co-workers proposed to use CeNPs for the treatment of ocular inflammation [20] while in 2020, Hyeon and co-workers have synthesized heterostructured CeO_2_/Mn_3_O_4_ nanocrystals for the protection of hematopoietic intestinal stem cells from irradiation-induced ROS damage [21].

Despite the very promising properties of metal nanoparticles as antioxidants [22], the environmental impact of their long-term use have been questioned, [23]. This problem can be attenuated using biodegradable nanoparticles. Lignin, as a polymeric polyphenol, is considered a promising natural antioxidant agent and several studies have been developed to explore this property [24,25]. However, it has a limited solubility in organic solvents, so various approaches are used to decrease its molecular weight and increase its content of –OH groups. Studies to obtain and characterize lignin nanoparticles are quite recent with only a few reports in the literature so far [26,27].

In 2018, Puglia et al. prepared spherical lignin nanoparticles by dissolving pristine alkali lignin into ethylene glycol, followed by addition of different acids. Higher radical trapping by lignin nanoparticles aqueous solution with respect of pristine lignin was revealed [28]. In 2019, Cheng et al. synthesized lignin nanoparticles by adding an anti-solvent to a colloidal dispersion of lignin in DMSO. They observed how the size of the nanoparticles and consequently, both the content and accessibility of phenolic hydroxyl group, affect the radical trapping activity of the latter [29]. Lastly, in 2020, Rezende and co-workers isolated pure lignin from a non-food biomass resource. These lignin nanoparticles also showed a radical trapping activity about 1.5 times higher than lignin in solution, and 3–4 times higher than butylated hydroxytoluene (BHT) and butylated hydroxyanisole (BHA), respectively, indicating their potential applicability as active antioxidant materials in dermocosmetics products [30].

Advancement in nanotechnology has revealed several nanoparticles with biological origins, such as melanin nanoparticles as potent antioxidants by themselves [31]. Melanins have been proposed to possess antioxidant activity [32,33], that has been related, for instance, to their anti-inflammatory [34], wound regeneration [35] and anti-ischemic activity [31]. We have recently explained the antioxidant properties of polydopamine (PDA) in the synergic trapping of alkylperoxyl and hydroperoxyl radicals. The key reaction explaining this peculiar antioxidant activity is the reduction of the *ortho*-quinone moieties present in PDA by the reaction with HOO^•^ [36].

Due to their native biocompatibility and their biodegradability, melanins are gaining increasing attention ranging from nanomedicine to nanocosmetics [37]. Inspired by PDA nanoparticles, nanoparticles based on the autoxidation of serotonin (5-hydroxytryptamine) and of 1,8-dihydroxynaphthalene (DHN) have been designed and developed, to obtain, respectively, polyserotonin and DHN allomelanin, a type of nitrogen-free melanin. Polyserotonin nanoparticles, obtained from oxidative polymerization of the well-known neurotransmitter serotonin, was used as promising nanomaterials for cancer therapeutics by testing its photothermal properties, drug loading and release, and biocompatibility [38]. Artificial allomelanin nanoparticles instead, showed radiation protection in human skin cells via radical scavenging [39].

Finally, another example of intrinsically active nanoantioxidants concerns carbon nanomaterials, whose structures are based primarily on sp^2^-hybridized carbon bonding, and include fullerenes [40], graphenes [41], carbon nanotubes [42] and their derivatives [43]. It was demonstrated that they exhibit strong ROS-scavenging properties [44]. Carbon nanomaterials possess such properties due to their conjugated π-system, which permits the scavenging of free radicals by addition to double bonds [45].

### 2.2. Functionalized Nanoparticles

The antioxidant properties of functionalized nanoparticles strictly depend on the compounds used to decorate the surface. The nanoparticle structure, in addition to acting as a support, may provide specific features to the final materials, that would be impossible to obtain with small-molecule antioxidants, such as magnetic properties. Surface functionalization has been typically performed by exploiting natural or synthetic antioxidants including glutathione [46], carotenoids [47], gallic acid [48], curcumin [49], rosmarinic acid [50], caffeic acid [51], α-tocopherol analogues [52], and BHT [53] but also biomacromolecules such as protamine sulfate polyelectrolyte (PSP) and SOD enzyme [54]. Recently, nanoantioxidants functionalized with surface-bound hindered nitroxides have also been proposed [55,56]. Nitroxides derived from TEMPO (2,2,6,6-tetramethylpiperidine 1-oxyl) are a class of persistent radicals characterized by high stability in water under air [57] that exhibit antioxidant activity and no in vitro toxicity and can be conveniently used to obtain different kinds of nanoantioxidants.

**Figure 2:**
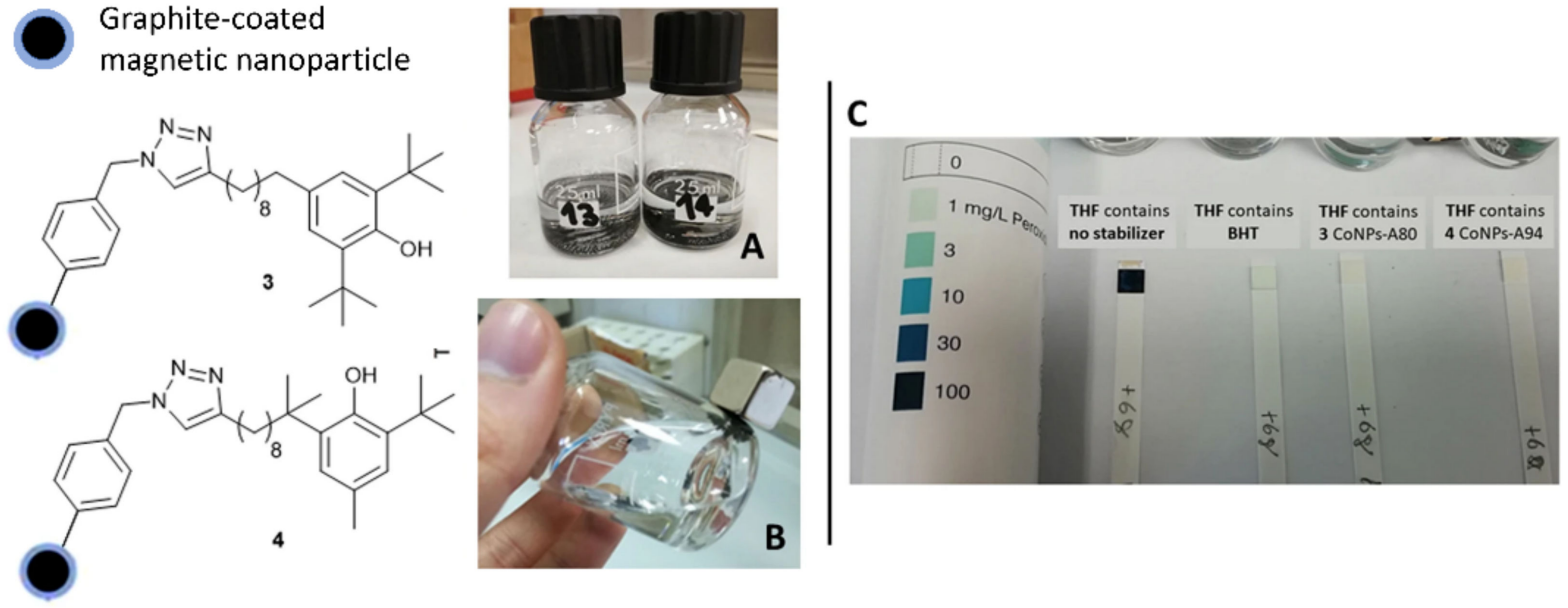
Nanoantioxidants **3** and **4** suspended in THF (inset A) and recovered with a neodymium magnet (inset B), peroxide test strips (QUANTOFIX Peroxide 100, inset C) reveal the absence of peroxide formation in samples containing the nanoantioxidants and the reference BHT. Adapted from Ref [5].

The surface functionalization of Fe_3_O_4_ nanoparticles with gallic acid allowed to obtain magnetically separable, efficient and low cost nanoantioxidant with potential applications in polymer, cosmetics, biomedical and food industry [58]. In 2019, magnetite-quercetin complex with free radical scavenging capacity and super-paramagnetic behaviour were obtained and showed potential antifungal and antibacterial effects [59]. Carbon-coated cobalt nanomagnets decorated by α-tocopherol units or BHT (see the example reported in Figure 2) are able to effectively counteract the autoxidation of organic substrates, and can be efficiently controlled by an external magnet [5,60].

On the other hand, nano-encapsulated materials allow for better stability and serve as carriers for the controlled release of the compounds they contain. This is the case of ascorbic acid in liposomes [61] and in porous silica nanoparticles [62]. Halloysite is a natural clay with an intrinsic nanotubular structure that has been used to load a diarylamine antioxidant for rubber stabilization [63], and various natural phenolic antioxidants such as curcumin [49], silibinin [64], resveratrol [65], and quercetin [52] to improve their stability and to obtain controlled antioxidant delivery systems.

The examples reported above demonstrate how research on nanoantioxidants is a trending topic that span very different fields. In most cases, however, the methods used to detect their antioxidant properties are not up to par with the other characterizations carried out on those types of materials, for example regarding the size of the nanoparticles, the percentage of loading or the release capacity. These latter data are obtained using appropriate and well-established techniques that rarely make inherent mistakes. Instead, regarding antioxidant properties, simplified methods such as the DPPH^•^ and ABTS^•+^ assays are almost exclusively used, whereas the ability of nanomaterials to block the autoxidation of organic substrates is only seldom investigated. In the next sections, we explain how to improve the understanding of our chemical knowledge on nanoantioxidants by using inhibited autoxidation experiments.

## 3. Why You Should Not Use Simplified Methods?

**Figure 3:**
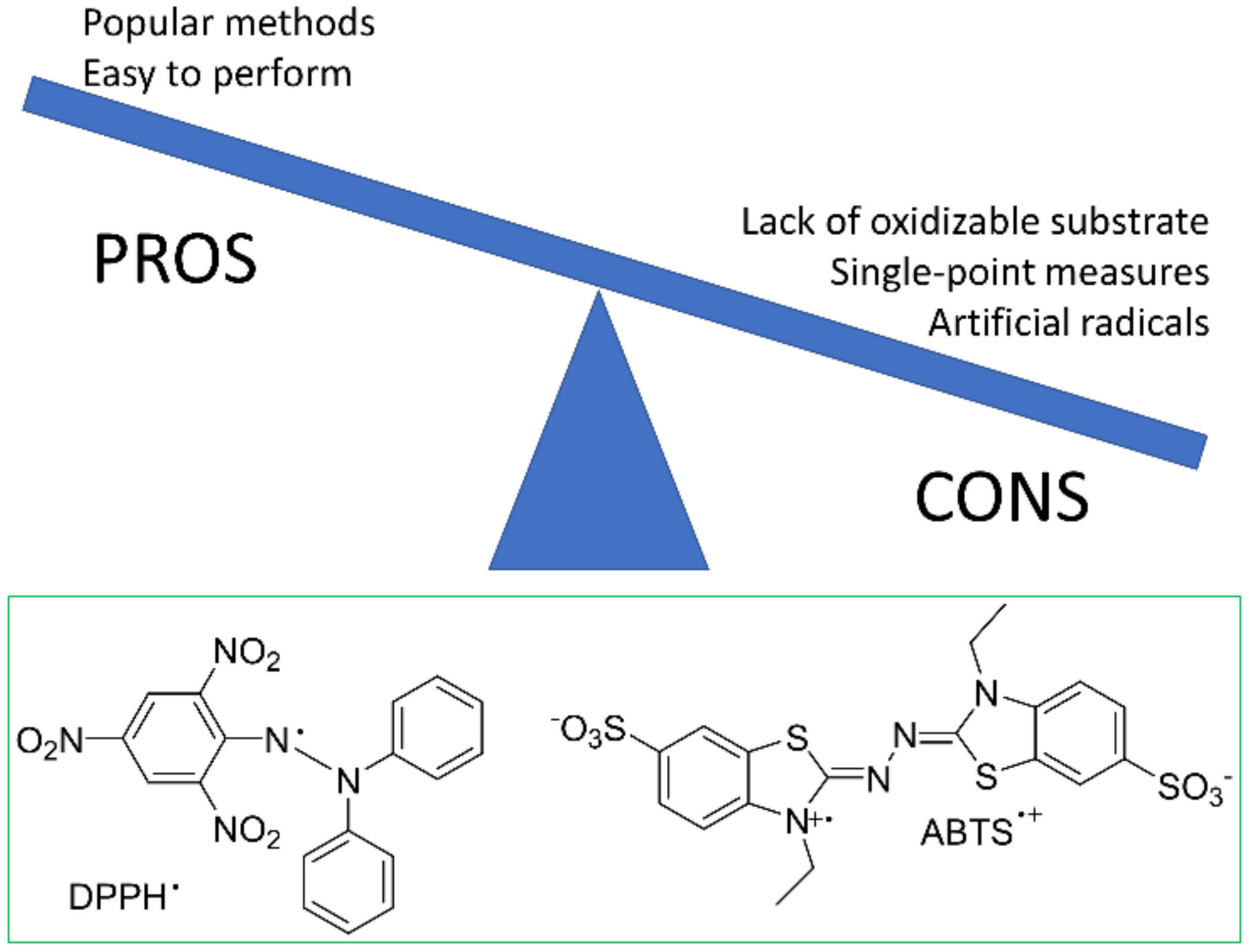
Structures of radical compound DPPH^•^ and ABTS^•+^.

Stable colored radicals such as DPPH^•^ and ABTS^•+^ (see Figure 3) are believed to be a simple way to measure the antioxidant activity, as their absorption changes upon reaction with reductants including antioxidants [66]. However, these assays have many limitations and their popularity may represent an obstacle to the development of nanoantioxidants, because they prevent researchers to investigate with more accuracy the reactions of biologically relevant radicals. The use of DPPH^•^ and related methods should be avoided especially in the field of new nanomaterials whose chemistry is still to be fully explored and we propose alternative techniques which can be easily implemented to replace these assays. Below, we list the main shortcoming of these techniques.

**Artificial radicals.** The stable radicals used as probes are chemically very different from the radicals that propagate the autoxidation of real systems, thus they don’t reproduce the kinetic and sometimes neither the stoichiometry of the reaction with ROO^•^. Moreover, these stable radicals may be quenched also by reductants having no antioxidant activity, such as H_2_O_2_ and hydroperoxides [66,67,68,69].

**Lack of oxidizable substrate.** The absence of a suitable oxidizable substrate makes impossible to know whether the putative antioxidant can interrupt the autoxidation of an organic molecule or not, or even display pro-oxidant effects. The results obtained indicate a “radical trapping power” or a “reducing power” rather than a true chain-breaking activity. The foregoing arguments are valid also for assays based on the reduction of metal ions (i.e., Ferric Reducing Antioxidant Power, FRAP, and CUPric ion Reducing Antioxidant Capaicty, CUPRAC assays) or of different kinds of stable radicals, such as galvinoxyl or dialkylnitroxides [70].

**Single-point measurements.** Besides these shortcomings that originate from the chemical structures of the stable radicals, other problems derive from single-point measurements of absorbance decay after a fixed time. The time interval employed is usually about 30 min (however, it may change from study to study), that is long enough to allow even low-activity antioxidants to react with the probes. These experiments therefore represent a sort of “titration” of the reducing groups present on a molecule or on a nanomaterial. On the other hand, the study of the kinetic decay of the probe, may provide a more realistic description of the reactivity, but is more difficult than single-point measures because it requires the kinetic analysis of the results, and a specialized instrumentation in case of fast reaction (such as stopped-flow equipment) [70].

**Optical interferences**. As the DPPH^•^ and related assays are based on spectrophotometric determinations, they may be affected by the scattering of the solution, or by the intense color of the materials that may be superimposed to the absorption maxima of the probes. These problems can be solved by using EPR spectroscopy as a detection method for the probe concentrations, or by separating the materials just before the absorbance reading [70].

## 4. Autoxidation Mechanism. Why Focus on Peroxyl Radicals?

### 4.1. Autoxidation

**Scheme 1:**
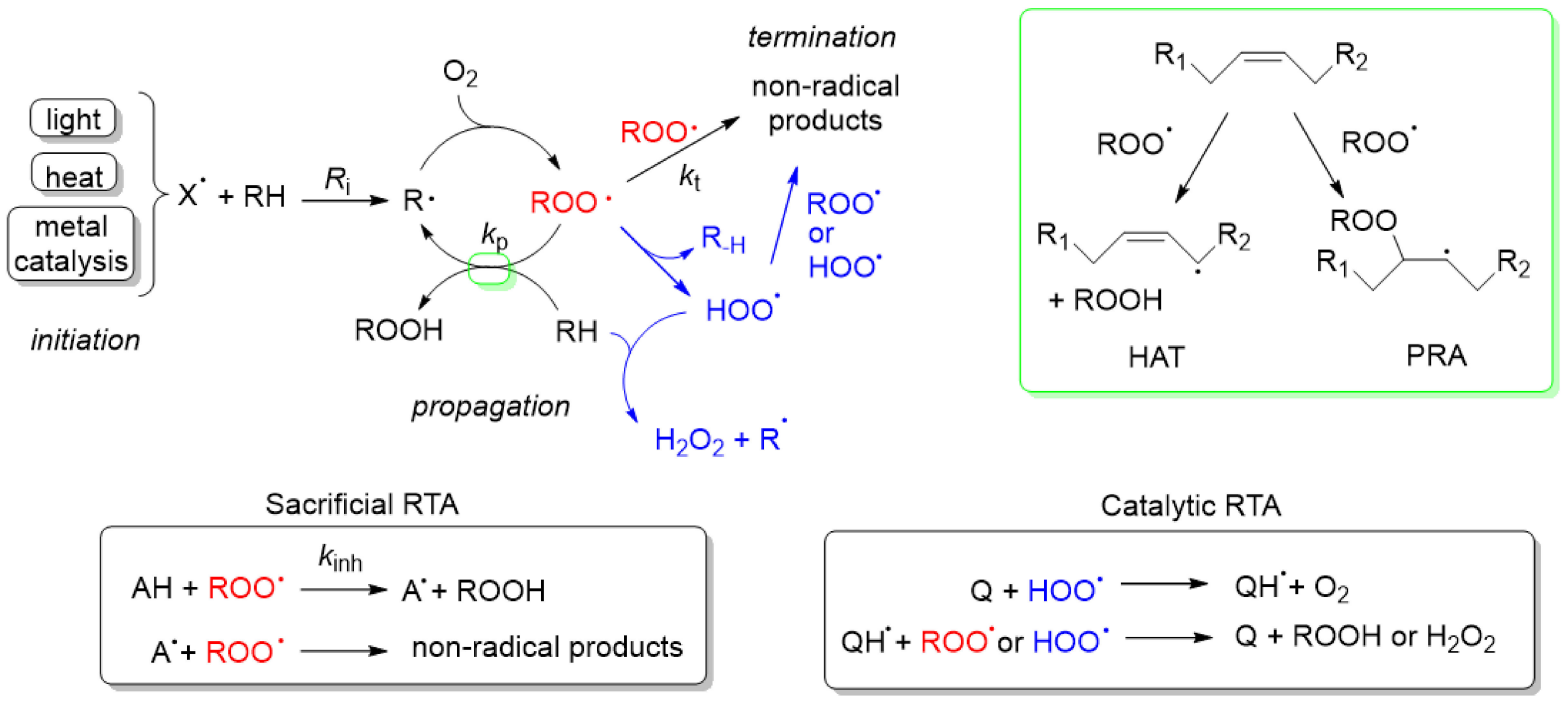
General mechanism of inhibited autoxidation. RTA = radical trapping antioxidants; HAT = H-atom transfer; PRA = peroxyl radical addition.

Autoxidation is the spontaneous reaction of organic molecules with O_2_, occurring through a radical-chain mechanism consisting of the typical initiation, propagation and termination steps depicted in Scheme 1 [1].

**Initiation**. Many different pathways can lead to the formation of radicals and thus contribute to the initiation step. UV and visible radiations are responsible for initiation in skin and materials exposed to light such as food and plastic, especially in the presence of pigments that may act as photosensitizers [71,72]. Temperature increase facilitates the endothermic reactions leading to radical formation, such as cleavage of weak bonds (such as RO–OR in peroxides) and the hydrogen atom transfer (HAT) from bis-allylic C–H groups to O_2_ [73]. These processes are important in cooking and long-term storage of unsaturated lipids. Metal catalysis is of paramount importance in water containing multiphasic systems, like living cells, food and cosmetic emulsions. The simultaneous presence of free iron and lipid hydroperoxides triggers cell death trough ferroptosis, and Fe^2+^ plus H_2_O_2_ cause the damage of biological macromolecules through the Fenton reaction [74]. Interfering with these processes is an important strategy to slow-down the autoxidation, for instance by reducing the exposure to light, heat, removing metals by chelation and decomposing H_2_O_2_. Some nanomaterials endow their antioxidant ability to one or more of these effects, and not to their ability to trap radicals. For instance, artificial melanin nanoparticles protects from light [75], and certain metal oxides can remove H_2_O_2_ behaving like the natural catalase (CAT) [76] or glutathione peroxidase (GpX) enzymes [77].

**Propagation**. The initiating radical X^•^ reacts with the oxidizable molecules forming a carbon-centred alkyl radical (R^•^) that in turn reacts with O_2_ to generate alkylperoxyl radicals (ROO^•^, see Scheme 1). As the reaction of R^•^ and O_2_ is very fast, very low levels of O_2_ are enough to quantitatively transform R^•^ to ROO^•^. Peroxyl radicals then propagate the oxidative chain by reacting with the substrate via HAT or peroxyl radical addition (PRA) to C=C double bonds, yielding a new alkyl radical (see the box in Scheme 1). The rate constant of the propagation reaction, *k*_p_, is the main determinant of the oxidation rate. In the case of pro-aromatic oxidizable substrates (i.e., γ-terpinene) or having alcohol or amine groups, the ROO^•^ radicals can undergo a 1,4-intramolecular HAT [78] leading to the formation of hydroperoxyl radicals (HOO^•^), which have a specific chemical behaviour that strongly influences the oxidation rate and the efficacy of antioxidants (see blue reactions in Scheme 1).

**Termination**. In the absence of antioxidants, radicals disappear by the self-recombination of two ROO^•^ radicals, while the role of R^•^ is negligible because the concentration of alkyl radicals, in the presence of O_2_, is very low. If HOO^•^ is formed, it speeds up termination thanks to the HAT reaction: HOO^•^ + ROO^•^ → ROOH + O_2_ [79].

### 4.2. Chain-Breaking or Radical-Trapping Antioxidants (RTA)

**Scheme 2:**
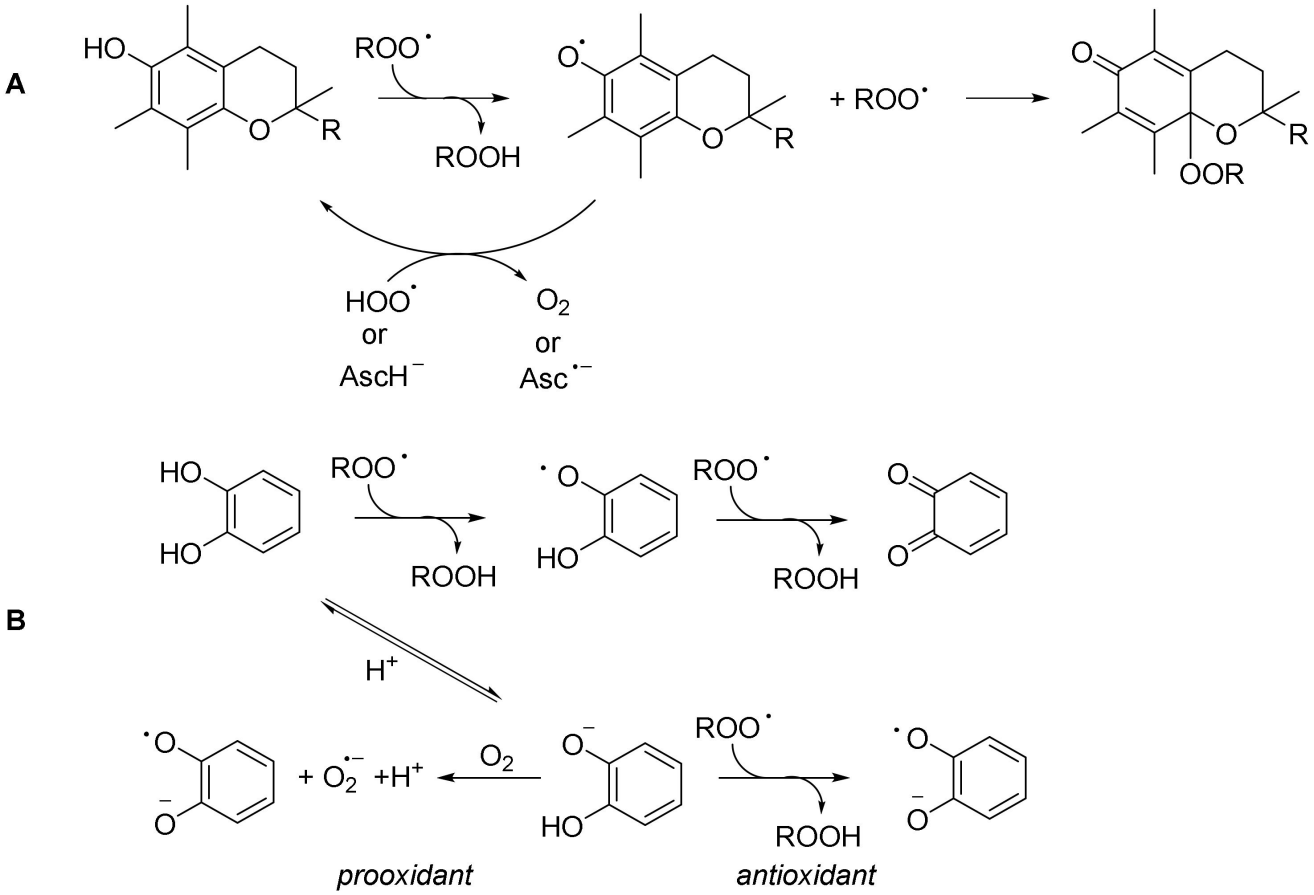
Typical mechanism of action of RTA. (**A**) Reaction of α-tocopherol (R = C_16_H_33_) with peroxyl radicals leading to a stoichiometry of 2 radicals trapped by each antioxidant. The mechanism of regeneration by ascorbate (AscH^−^) or by HOO^•^, leading to radical trapping stoichiometries larger than 2, is also shown. (**B**) Reaction of catechols with peroxyl radicals, and effect of the deprotonation of one OH group on the antioxidant/prooxidant behaviour of catechols.

This type of compounds blocks the autoxidation of organic molecules by quenching ROO^•^ radicals and forming stable radicals that don’t propagate the oxidative chain (Scheme 1). The rate constant for the reaction with ROO^•^, also called the inhibition rate constant, *k*_inh_, and the number of ROO^•^ trapped (i.e., the stoichiometric coefficient *n*) are the two parameters that describe the efficacy of an antioxidant. The great majority of the antioxidants act as sacrificial reductant toward ROO^•^, so they are consumed during the RTA activity, unless they are reduced back by other sacrificial reductants like in the case of the well-known synergy between α-tocopherol and ascorbate (AscH^−^) [80], or in the presence of HOO^•^ (see Scheme 2A) [81]. In the presence of HOO^•^, nitroxides and *ortho*-quinone [36] containing molecules (indicated with **Q** in Scheme 1) behave as catalytic antioxidants causing the disappearance of ROO^•^ and HOO^•^ with little antioxidant consumption [82,83]. The reaction between an RTA antioxidant and ROO^•^ can occur through a variety of sequential or concerted proton-coupled electron transfer mechanisms that are important to rationalize the antioxidant effect in any given reaction medium [84,85,86]. In general, antioxidants having a low bond dissociation enthalpy of the O–H or N–H bonds or low redox potential react quickly with ROO^•^ but at the same time are susceptible to react with O_2_. As the latter reaction causes the depletion of antioxidants over time, a compromise must be achieved between stability under O_2_ and reactivity toward ROO^•^. In the case of antioxidants based on a pyridine or pyrimidine nucleus, the reaction with O_2_ is minimized while conserving a good ROO^•^ trapping [87]. In this regard, the deprotonation of antioxidants (such as ascorbic acid or polyphenols) greatly increases their reaction with ROO^•^, but also decreases their stability [88,89] (Scheme 2B).

### 4.3. Why Focus on Peroxyl Radicals?

While a variety of radicals can be transiently formed during the autoxidation of organic substrates (see Table 1), only alkylperoxyl and hydroperoxyl radicals are important for the action of RTA antioxidants.

As mentioned before, alkyl radicals even under low O_2_ concentrations are promptly converted into ROO^•^, thus they are not intercepted by RTA. Hydroxyl (HO^•^) and alkoxyl (RO^•^) radicals are too reactive toward any organic substrates to be trapped by RTA. Actually, the antioxidant effect of RTA arises from the competition between the reaction of the radical with the substrate and that with RTA, thus if the former reaction is too fast, exceedingly high concentrations of RTA would be needed to stop this reaction [70]. The assays studying the reaction with HO^•^ are therefore not recommended as unique methods to assess the antioxidant activity.

### 4.4. SOD-like Activity

**Figure 4:**
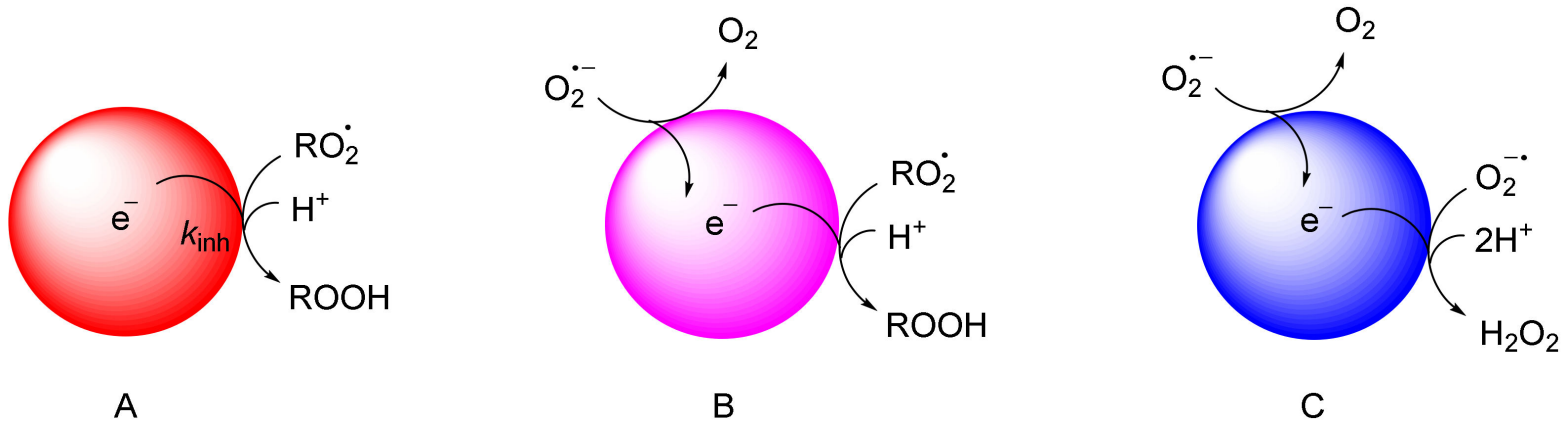
Similarities and differences between sacrificial (**A**), catalytic (**B**) and SOD-like nanoantioxidants (**C**). The reactions are exemplified for the case of nanoparticles that are only able to donate or accept electron, with protons being released by the solvent. However in many case nanoparticles can host protons on the surface thus displaying a behaviour identical to that of phenols or of quinones.

Superoxide (O_2_^•−^) is not reactive toward organic substrates and thus it does not propagate the oxidative chain, and it is also unreactive toward most RTA [90]. Nevertheless, O_2_^•−^ is a precursor of H_2_O_2_, that is a non-radical ROS (reactive oxygen species) which influences cellular functions and may originate HO^•^ radicals. Certain nanomaterials, in particular metal oxides (CeO_2_, Mn_3_O_4_, …) have superoxide dismutase (SOD)-like activity because they are cyclically reduced and oxidized by O_2_^•−^ as shown in Figure 4 [91] and display interesting biological effects [76].

## 5. How to Study Autoxidations Inhibited by Nanoantioxidants?

The best methods to measure the antioxidant activity of RTA are based on the ability to slow down the autoxidation of an organic substrate, under conditions that should be as similar as possible to those occurring in real systems. For instance, if the antioxidant is intended for food applications, the oxidizable substrate may be represented by polyunsaturated fatty acids or by their esters, as bulk oils or as oil/water emulsions. Instead, if biological applications are sought, liposomes made of unsaturated phospholipids may be used. It should be noticed that many antioxidants (especially polyphenols) [89] become pro-oxidants at high concentrations, thus the dependency of the chain-breaking activity on concentration should be assessed. Autooxidation-based methods are able to detect conditions at which a given antioxidant nanomaterial can act as pro-oxidant. However, although autoxidation studies are a well assessed method, their application to nanoantioxidants is not widespread and thus some guidelines are presented in this section.

### 5.1. Initiation

**Figure 5:**
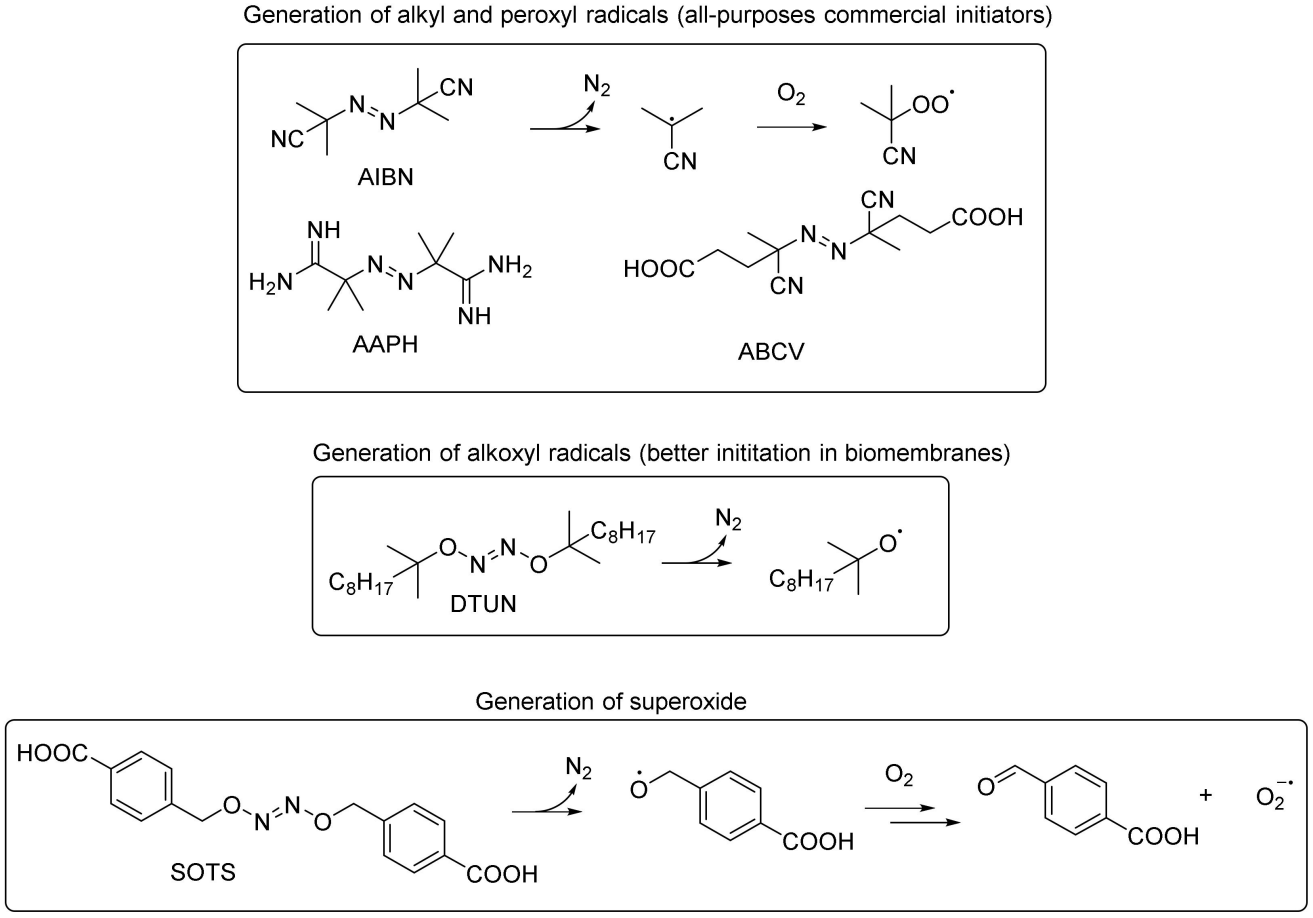
Different class of azoinitiators currently used in antioxidant research.

As spontaneous autoxidation at room temperature is a slow process that takes several days to proceed, accelerating strategies are usually employed. In food and biodiesel research, autoxidation is accelerated by rising the temperature to 90–130 °C, such as in the Rancimat and similar OSI (oxygen stability index) methods [92]. Although temperature is a “clean” expedient, as it doesn’t require the addition of initiating compounds, it has many drawbacks, including the volatilization of low-boiling components, that makes difficult the extrapolation of the results to the room temperature. Small amounts of chemical initiators, such as azoinitiators or peroxides, are the most practical way to make autoxidations fast and reproducible at room temperature. Azoinitiators with different solubilities and decomposition rates are commercially available (Figure 5) and can be conveniently used to generate radicals at a constant rate, which is a fundamental prerequisite to perform meaningful autoxidation studies. Initiator consumption is usually negligible at low temperatures, so their concentration can be considered constant during the reaction [93]. Novel initiators have been proposed for specific applications, such as to initiate the autoxidation of liposomial bilayers, or to study the chemistry of superoxide (Figure 5) [94]. Another initiation strategy includes the use of Fe^2+^ with peroxides (usually H_2_O_2_) although in this case a constant initiation rate can hardly be achieved because Fe^2+^ is quickly consumed, while the level of peroxides increases during the autoxidation [95].

### 5.2. Oxidizable Substrates and Reaction Medium

**Figure 6:**
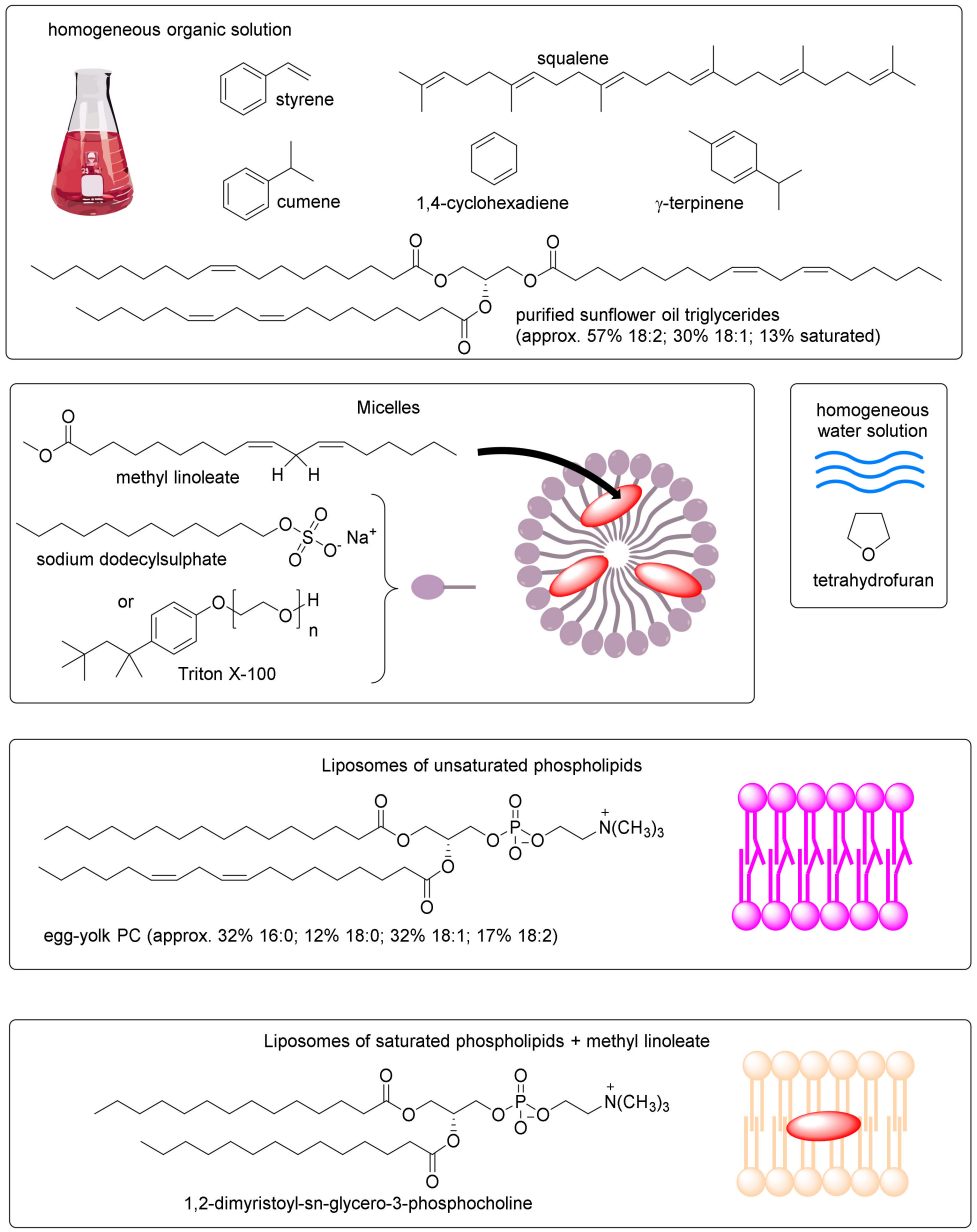
Oxidizable substrates for inhibited autoxidation studies (PC = phosphatidylcholine).

The choice of the oxidizable substrate is also fundamental to obtain meaningful results from autoxidation studies. If quantitative chemico-physical determinations are requested (i.e., measure of kinetic rate constants, see Section 5.5), well-characterized substrates must be chosen. For instance, the propagation and termination rate constants are known (mostly at 30–37 °C) for some organic molecules in homogeneous solution, such as methyl linoleate, styrene, cumene, tetrahydrofuran, sunflower oil triacylglycerides, squalene and para-cymene (see Table 2). Experiments in homogeneous water solution can be performed by using water-soluble oxidizable substrates (such as tetrahydrofuran, THF). Micelles can be used to obtain micro-heterogeneous systems by using neutral or charged surfactants (typically sodium dodecylsulfate [96] or Triton X-100 [97]) and methyl linoleate as substrate. In order to mimic cell membranes, liposomes made of phospholipids containing polyunsaturated fatty acids (such as egg yolk phosphatidylcholine) are the best choice [94]. Another model can be represented by saturated phospholipids incorporating methyl linoleate (see Figure 6) [98].

### 5.3. How to Study an Autoxidation?

**Figure 7:**
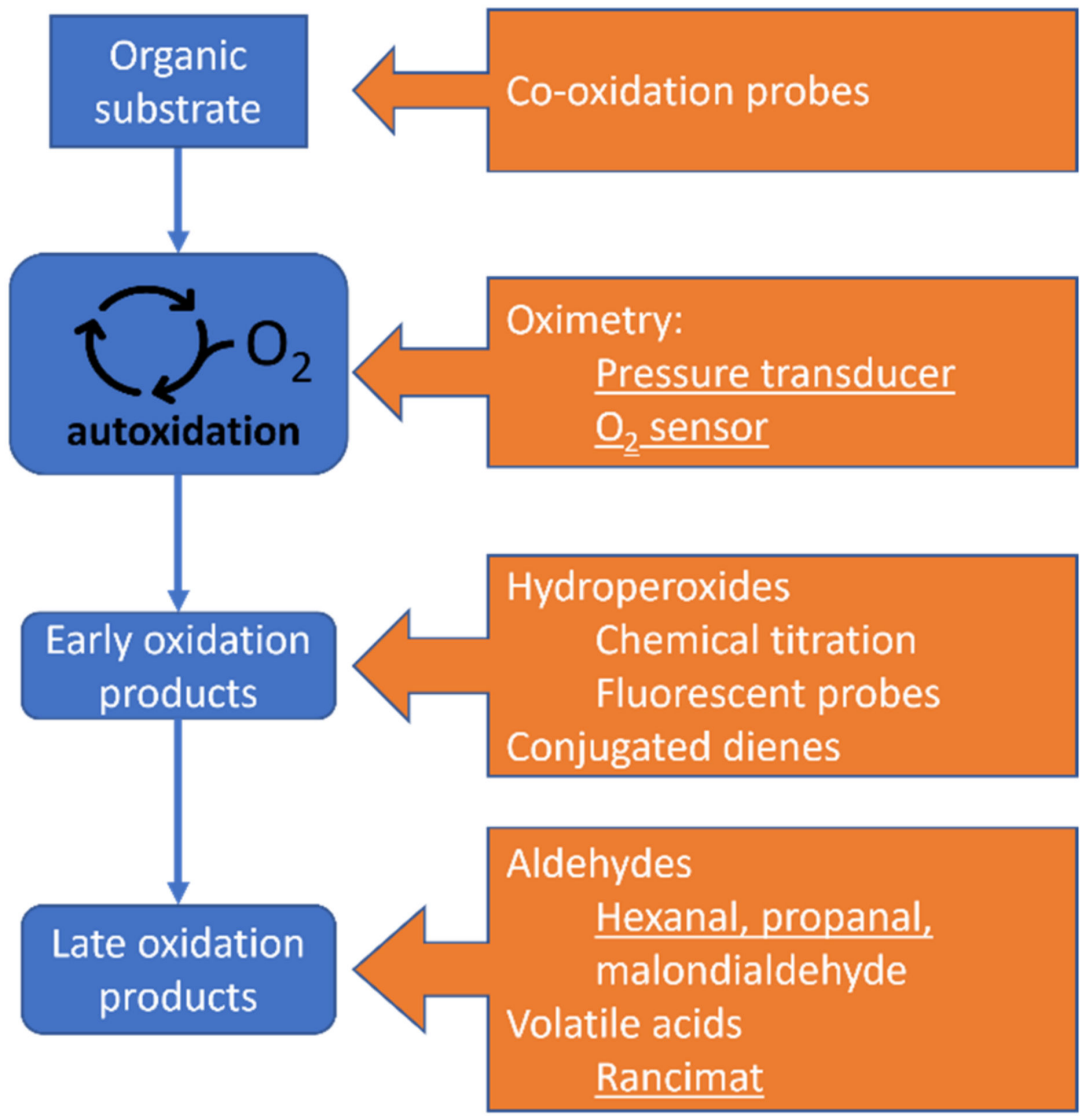
Methods (in orange boxes) used to study the rate of autoxidation. The methods that are not based on optical spectroscopy and thus are best suited to study nanoantioxidants are evidenced.

The autoxidation of a given substrate can be studied by the disappearance of reactants or the formation of products, that in turn can be differentiated into early and late products (see Figure 7).

#### 5.3.1. Disappearance of Reactants

**Scheme 3:**
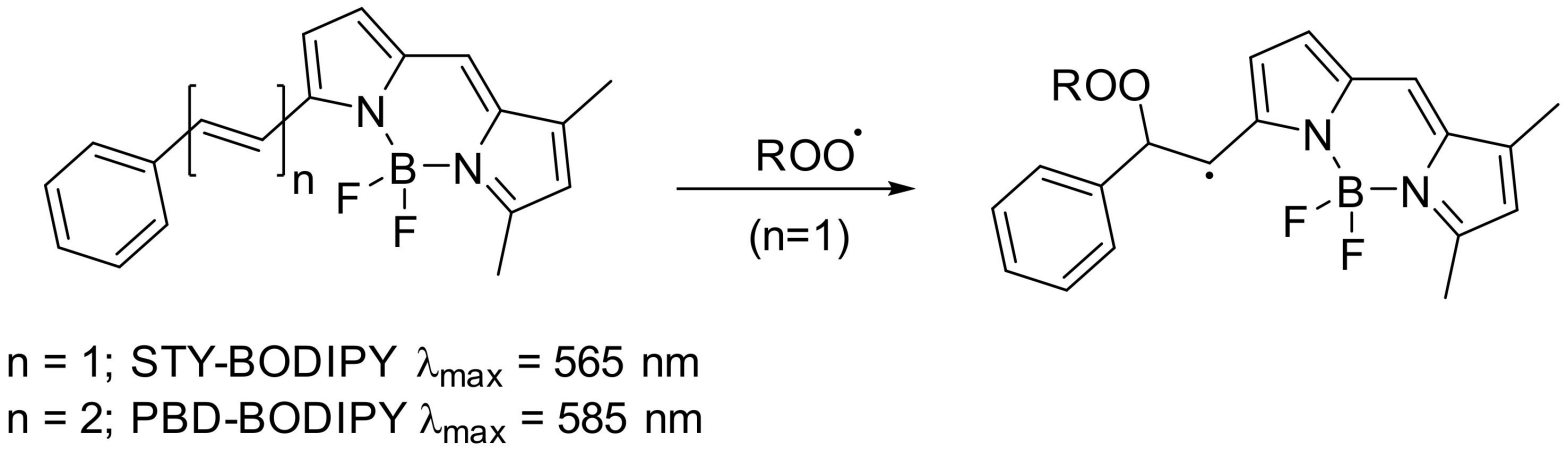
Probes used to study autoxidation reactions.

Oxygen consumption is arguably the process that unifies the oxidation of many different substrates, and oximetry is the most versatile method that can be applied to a variety of samples, including nanomaterials, which are not suitable for HPLC analysis or optical spectroscopy. Given their importance, the techniques for measuring O_2_ during autoxidation are described in detail in the next section. Another method to monitor the disappearance of reactants consists of using probes having an intense absorption (or fluorescence emission) and a chemical structure that resembles that of oxidizable substrates, such as STY-BODIPY or PBD-BODIPY (Scheme 3) [106]. The probes can be co-oxidized with the substrate and followed by UV-vis or fluorescence spectroscopy. The advantage of this method is its high throughput as it can be implemented for use in thermostatic microplate readers.

#### 5.3.2. Formation of Early Products

Hydroperoxides are the most common early products formed during an autoxidation, however it should be emphasized that certain substrates may preferentially form other oxygenated products such as dialkylperoxides or epoxides, and that hydroperoxides are unstable especially at high temperature and in the presence of acids or metals. Hydroperoxides can be determined by iodometric titration, by the ferrous thiocyanate assay or by using fluorescent probes [107]. In the case of the autoxidation of polyunsaturated fatty acids, the hydroperoxides possess conjugated dienes that can be evidenced by spectrophotometry or HPLC-UV-vis detection [108,109]. The analysis of the regiochemistry of the hydroperoxides of linoleic acid or of simpler alkenes also can provide information about the activity of antioxidants (the so called peroxyl-radical clock) [110].

#### 5.3.3. Formation of Late Products

The products that arise from the decomposition of hydroperoxides mainly include aldehydes and acids. Hydroperoxide fragmentation is induced by heat or by the acid-catalysed Hock transposition. The latter reaction can occur during the autoxidation by the traces of acids present in the solvent, or more frequently it is induced by addition of acids during the preparation of the samples [111]. Among the various aldehydes formed by the decomposition of hydroperoxides of polyunsaturated lipids, malondialdehyde is particularly important because its levels are detected by the popular TBARS (thiobarbituric acid reactive substances) method, which is based on the detection of its coloured adduct with thiobarbituric acid. The many limits of the TBARS assay have been reviewed and, due to its low specificity, TBARS method is not recommended for determination of malondialdehyde as one of late product of lipid peroxidation [112]. Low-molecular-weight acids are detected by the Rancimat method, that is based on fluxing an air stream in a heated lipid sample in the presence of the antioxidant, checking the formation of volatile products by bubbling the gases into purified water and measuring the conductivity of this solution [92]. Hexanal and propanal can be conveniently measured in the headspace of the reaction vessel by a gas chromatograph equipped with a solid phase micro extraction (SPME) injector [107].

### 5.4. Advantages of Oximetry

Most autoxidation methods are not easily applicable to the study of nanoantioxidants. Assays relying on spectrofluorimetry or spectrophotometry may suffer from the light scattering displayed by many nanomaterials, from the intense colors caused by plasmon resonance in metal nanoparticles or from the absorption of highly conjugated materials such as melanins and carbon-based nanomaterials. In this context, the study of the autoxidation by measuring O_2_ consumption has no limitation respect the use of these kinds of nanoantioxidants, because there is no optical interaction with the sample.

O_2_ consumption can be measured by using two different approaches: (i) measure of the pressure drop due to O_2_ consumption, that can be achieved by different kinds of pressure gauges and (ii) direct measure of O_2_ concentration, either in water or in the headspace, by O_2_-sensitive probes. Differential pressure transducers measure the small pressure differences between a sample and a reference reaction flask and can be applied to both organic solvents and aqueous solutions. The reference flask contains the same reaction mixture as the sample, but in the presence of a high concentration of an antioxidant, so that it allows to correct the pressure drop observed during the autoxidation of the substrate for the N_2_ development and O_2_ consumption from the azo-initiator [93].

The direct measure of O_2_ concentration comprises optical or electrochemical O_2_ sensing. The first method is based on the fluorescence quenching, caused by O_2_, of a fluorescent probe attached on the tip of an optical fiber immersed in the sample or inserted in the headspace. This equipment works best in air and in water but is incompatible with organic solvents [89,113]. A second method suitable for aqueous systems is based on a polarographic Clark electrode, that produces a current that is proportional to the amount of O_2_ that reaches the electrode tip after crossing a polymeric membrane [114].

### 5.5. Kinetic Analysis of O_2_ Consumption Plots

The oxygen consumption during the autoxidation of a generic substrate RH, in the absence of antioxidants, is described by Equation (1), where *k_p_* and *k_t_* are, respectively, the propagation and termination rate constant for the autoxidation of the oxidizable substrate and *R_i_* is the initiation rate.

−*d*[O_2_]/*dt* = (*k_p_*/√2*k_t_*) [RH] √*R_i_* + *R_i_*, (1)

Some examples of typical oxidizable substrates are given in Table 2, together with their *k_p_* and 2*k_t_* values. From the values reported herein, it is evident that the *k_p_* constant varies considerably, from very low values for saturated hydrocarbons to 10^4^-fold higher values for polyunsaturated hydrocarbons. As 2*k_t_* values with a few notable exceptions (see for instance isopropyl benzene) are in the range 10^6^–10^7^ M^−1^ s^−1^, the main determinant of a substrate oxidation is the propagation constant.

The oxygen consumption in the presence of a RTA is described by Equation (2) in the assumption that every ROO^•^ radical is trapped by AH and A^•^, while the duration of the inhibition period (*τ*) is given by Equation (3), where *n* is the stoichiometric coefficient, that is the number of radical trapped by each molecule of antioxidant.

−*d*[O_2_]_inh_/*dt* = (*k_p_*[RH]*R_i_*)/(*nk*_inh_[AH]) + *R_i_*, (2)

*τ* = ([AH]*n*)/*R_i_*, (3)

Equation (3) provides an easy mean to measure *R_i_*, by using a reference antioxidant with a known *n* value. For this purpose, usually α-tocopherol and its synthetic derivatives are used, as they trap two radicals (*n* = 2) (see Scheme 2A).

**Figure 8:**
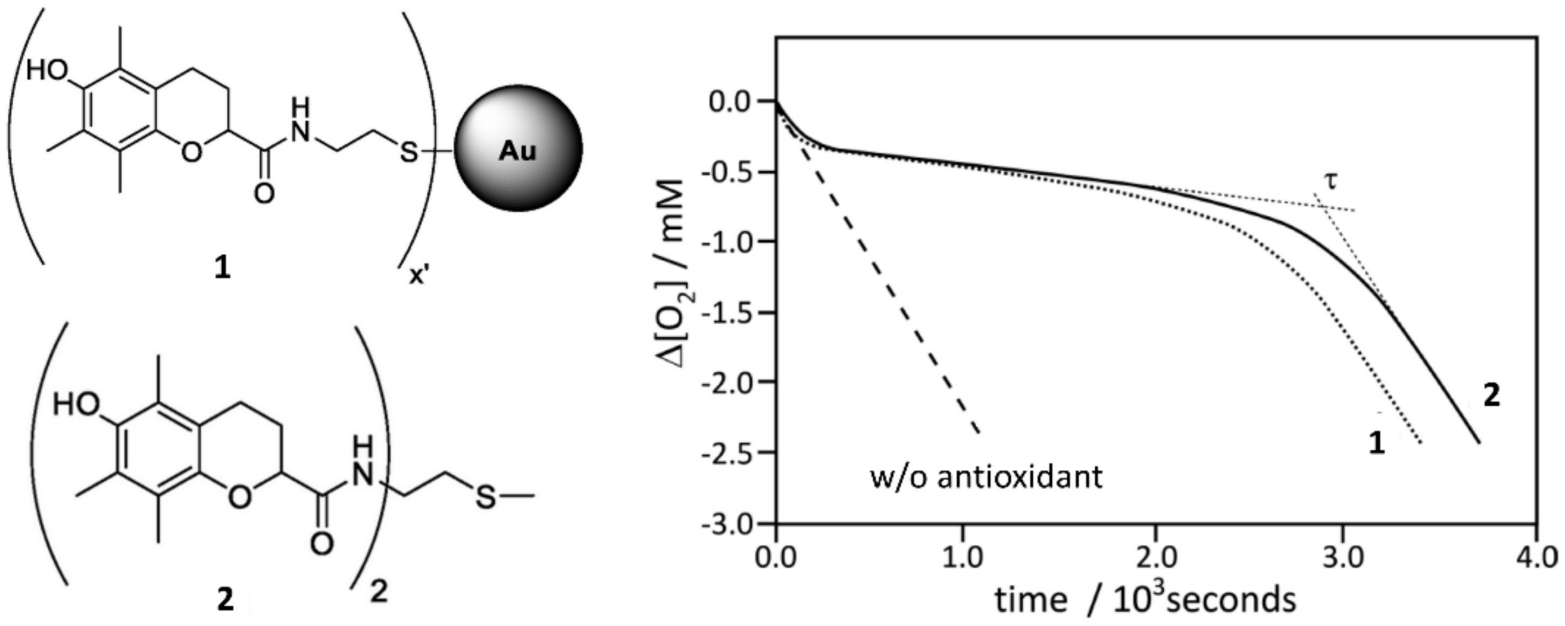
O_2_ consumption traces observed during the autoxidation of styrene 4.3 M in chlorobenzene initiated by 50 mM of AIBN in a sample volume 4 mL at 30 °C, inhibited by nanoparticles bearing an analogue of α-tocopherol on the surface (**1**, 10.3 mg/mL) and by the antioxidant dimer (**2**, 5 μM). The O_2_ consumption was measured by a pressure transducer. Adapted from reference [98].

The effectiveness of an antioxidant is therefore described by two independent parameters: the inhibition rate constant *k*_inh_ and the number of radical trapped *n*. From Equation (2) it is evident that the autoxidation of substrates with a high *k_p_* is slowed down with more difficulty by antioxidants. A good inhibition is achieved only by antioxidants having a high *k*_inh_ or that are present in high concentration in the sample. Styrene, with *k_p_* of 41 M^−1^ s^−1^, is used to measure *k*_inh_ values in the range 10^5^ –10^7^ M^−1^ s^−1^, while cumene (*k_p_* = 0.32 M^−1^ s^−1^) is better suited for *k*_inh_ values in the range 10^3^ –10^5^ M^−1^ s^−1^. The autoxidation experiments in the presence of weak antioxidants, that provide only a retardation of the O_2_ consumption, require a specific kinetic treatment as in these cases self-termination (2 ROO^•^ → products) cannot be neglected (see reference [93] for details). As a general rule, autoxidation should be performed in solvents that are not reactive toward peroxyl radicals, such as chlorobenzene and acetonitrile (Figure 8). The presence of traces of acids or bases that could catalyse the reaction of ROO^•^ radicals with antioxidants must be carefully controlled [84]. Moreover, O_2_ consumption in every part of the autoxidation must be a few times bigger than *R*_i_, to ensure that the trapped ROO^•^ radicals are from the substrate and not from the initiator, and to avoid that *k*_inh_ value is underestimated.

Equation (2) has been used by our research group on several occasions to determine the *k*_inh_ of nanoantioxidants, as reported in Table 3, in the case of small antioxidants covalently bound on the surface of nanomaterials, or adsorbed into nanotubes or nanosponges. When a clear inhibition of the autoxidation can be detected, the antioxidant loading can be obtained by the duration of the inhibition time, in the assumption that the stoichiometry of inhibition is the same as that of the parent antioxidant.

### 5.6. Catalytic Antioxidants

**Figure 9:**
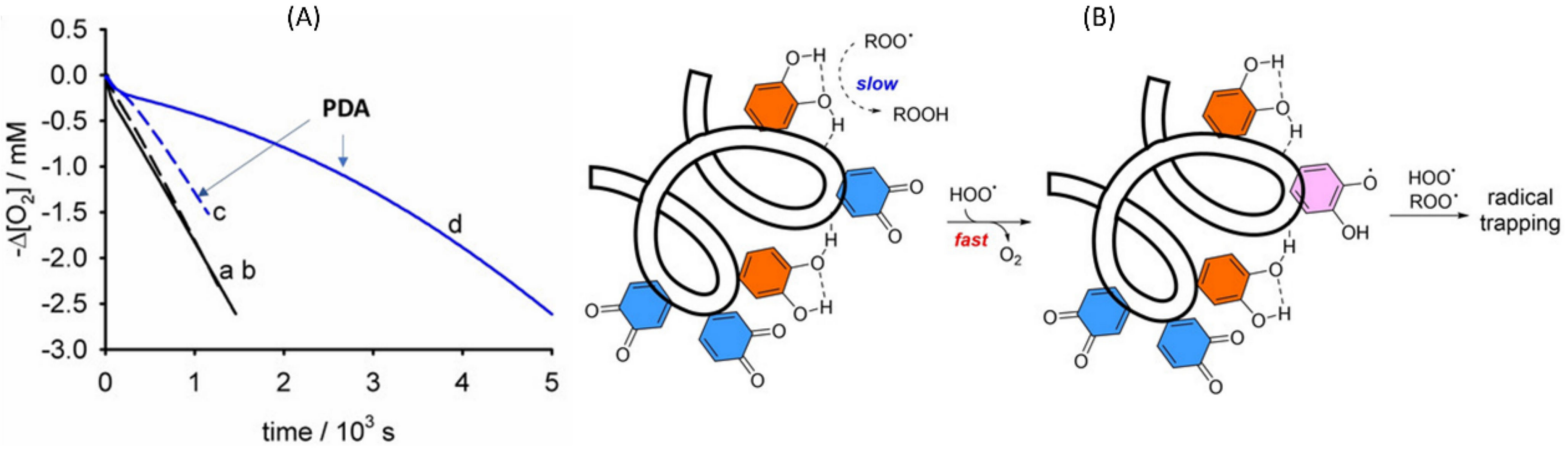
Catalytic antioxidant activity of polydopamine (PDA). Panel (**A**): O_2_ consumption during the autoxidation of styrene (initiator AIBN, 30 °C) without any inhibitor (a), with 1,4-cyclohexadiene (CHD) as source of HOO^•^ radicals (b), with PDA (c), in the presence of both PDA and CHD (d). Panel (**B**): mechanism explaining the radical trapping activity of PDA showing the reduction of quinone moieties to semiquinone able to trap new radicals by cycling between the quinone/semiquinone/hydroquinone forms. The O_2_ consumption was measured by a pressure transducer. Adapted from reference. [36].

Some antioxidants display a very long inhibition period that goes much beyond the value expected from the concentration of the antioxidant and *R*_i_, indicating an *n* value larger than 2, that is the typical value for phenolic antioxidants (see Scheme 2). The first explanation is that the antioxidant is alternatively oxidized and reduced by the radicals formed during the autoxidation and thus it facilitates the self-termination of radicals without being consumed. Typically, this effect is observed when the autoxidation of the substrate proceeds through the formation of HOO^•^ or mixed ROO^•^/HOO^•^ radicals [83]. Recent studies have shown that quinones and quinone-containing nanomaterials such as polydopamine show catalytic antioxidant activity by being cyclically reduced by HOO^•^ and oxidized by HOO^•^ or ROO^•^ [36] (Figure 9). Another example is given by the dialkyl nitroxide TEMPO, which catalytically reacts in organic solvents with ROO^•^ and HOO^•^ by a reductive cycle [82]. To investigate this property, specific substrates able to generate HOO^•^ such as 1,4-cyclohexadiene or γ-terpinene can be used, alone or in mixture with “ordinary” substrates that oxidize through ROO^•^ radicals.

A second explanation for the catalytic antioxidant effect is the regeneration of the oxidized form of the antioxidant by a sacrificial reductant without producing new radicals. This peculiar property has been discovered in the case of nitroxides in water, as in this solvent nitroxides are oxidized by ROO^•^ to the oxoammonium cation, that is reduced back to hydroxylamine by a hydride transfer from the substrate (such as THF) [115].

### 5.7. Studying Pro-Oxidant Activity

Prooxidant activity is an important aspect of the chemistry of nanomaterial related to their redox properties that is at the basis of their toxicity and antitumoral activity [116]. Interestingly, by performing experiments in the absence of the initiator, oximetry can be used to easily investigate pro-oxidant activity of nanomaterials. For instance, octanethiol-capped gold nanoparticles were found to trigger the autoxidation of an organic substrate (styrene) because the traces of tetra octyl ammonium bromide used as phase transfer agent in the synthesis promoted the homolytic decomposition of organic hydroperoxides (*^t^*BuOOH). The rate of generation of radicals by the system Au-NP/*^t^*BuOOH could be determined by the standard addition of a reference antioxidant [117].

### 5.8. Limitations of Oximetry Methods

Despite the advantages listed above, the study of autoxidations by oximetry methods is not widespread. The first reason is that autoxidations are slow reactions and therefore these methods are more time consuming than, for instance, those based on stable radical quenching. The second reason is that measuring O_2_ consumption requires specialized instrumentation, that is not present in most laboratories, although it is not particularly expensive. For instance, the pressure transducer equipment, used to perform the experiments reported in Figure 8 and Figure 9, is not commercially available, but it can be easily built from its parts at an affordable price for most laboratories [118]. Optical O_2_ probes, that are suitable for O_2_ determinations in gas and H_2_O, [113] have a similar cost.

## 6. Conclusions

This review highlights the progress made in recent years in the understanding of the mechanism of action of nanomaterials with antioxidant activity, and in the chemical methods used to evaluate their potency. It is demonstrated that inhibited autoxidation experiments provide valuable information about the interaction with the most important radicals involved in the lipid oxidation, namely alkylperoxyl and hydroperoxyl radicals. Autoxidation methods should complement (and possibly replace) the use of assays based on the quenching of stable radicals (such as DPPH^•^ and ABTS^•+^), because these are artificial radicals that are different from those occurring in real oxidation systems. Based on these considerations a checklist for the rational development of nanoantioxidant can be proposed.

(1) Is the nanomaterial able to stop the autoxidation of a relevant oxidizable substrate?
(2) What is the *k*_inh_ of the nanoantioxidant?
(3) What is the duration of the inhibition period? Does it behave in a catalytic fashion?
(4) Is the nanoantioxidant stable under the reaction conditions? Is it stable under air? Does it have a pro-oxidant effect the presence of alkyl and hydrogen peroxides?

Of course, for a practical use on nanoantioxidants, this list must be integrated with toxicological and environmental considerations [119], that go beyond the aims of this review [120]. Nevertheless, we believe that the use of these guidelines will inspire the research of novel nanomaterials able to cope with the autoxidation of organic materials in fields spanning from foods to plastic to biological systems.

## Figures and Tables

**Table 1:** Probability that radical-trapping antioxidants (RTA) intercept different types of radicals.

	Radical	Trapped by RTA	Reason
HO^•^	Hydroxyl	No	Reaction with organic substrates is too fast
RO^•^	Alkoxyl	No	Reaction with organic substrates is too fast
R^•^	Alkyl	No	Reaction with O_2_ is diffusion-controlled and [O_2_] ≈ 10^−4^–10^−3^ M
ROO^•^	Alkyl peroxyl	Yes	Reaction with organic substrate is slow
HOO^•^	Hydroperoxyl	Yes	Reaction with organic substrate is slow
O_2_^•−^	Superoxide	No (Yes) ^1^	No reaction with organic substrates nor with most RTA

^1^ Superoxide is trapped by SOD and SOD-like antioxidants.

**Table 2:** Examples of oxidizable substrates with their *k_p_* and 2*k_t_* values (M^−1^ s^−1^). Temperature 30 °C.

Substrate	*k_p_*	2*k_t_*	Solvent	Reference
Styrene	41	4.2 × 10^7^	PhCl	[99]
Cumene	0.32	4.6 × 10^4^	PhCl	[100]
Squalene	0.68	7.4 × 10^6^	PhCl	[101]
1,4-Cyclohexadiene	1400	1.3 × 10^9 a^	PhCl	[102]
*p*-Cymene	0.83	2.9 × 10^6^	PhCl	[101]
Stripped sunflower oil	66.9	3.5 × 10^6^	PhCl	[101]
Methyl linoleate	62	8.8 × 10^6^	PhCl	[99]
	36	3.5 × 10^5^	Triton X-100 micelles	[103]
	41	0.6 × 10^6^	DMPC ^b^ liposomes	[104]
Tetrahydrofuran	4.8	6.6 × 10^7^	H_2_O	[105]

(^a^) 2*k_t_* strongly depends on solvent. (^b^) 1,2-dimyristoyl-sn-glycero-3-phosphocholine.

**Table 3:** Inhibition rate constants (*k*_inh_/M^−1^ s^−1^) of nanoantioxidants and of related small-molecule antioxidants (*k*_inh_’).

Nanoantioxidant	*k*_inh_	*k*_inh’_	Solvent	Reference
Trolox-HNT	(1.1 ± 0.1) × 10^6^	(9.8 ± 0.5) × 10^5^	Cumene/PhCl	[52]
TroloxS-AuNPs	(6.9 ± 0.4) × 10^5^	(6.9 ± 0.3) × 10^5^	Styrene/PhCl	[98]
CoNPs-Trolox	(5.6 ± 1.5) × 10^6^	(6.4 ± 1.0) × 10^5^	Styrene/PhCN	[60]
Ascorbic Acid/HNT	(1.4 ± 0.3) × 10^6^	(1.7 ± 0.2) × 10^6^	THF/Water	[3]
Nitroxides NPs	(1.5 ± 0.4) × 10^5^	(5.1 ± 1.5) × 10^6^	THF/Water	[56]
Curcumin-HNT	(1.7 ± 0.1) × 10^4^	(1.8 ± 0.2) × 10^4^	Cumene/PhCl	[49]
